# Causal association between allergic diseases and short stature: a mendelian randomization study revealing the potential mediating role of inflammatory proteins

**DOI:** 10.1016/j.jped.2026.101612

**Published:** 2026-09-18

**Authors:** Wei Yin, Liping Wang

**Affiliations:** Department of Pediatrics, Linping Branch, the Second Affiliated Hospital of Zhejiang University, Linping District, Hangzhou City, Zhejiang Province, China

**Keywords:** Allergic diseases, Short stature, Inflammatory proteins, Mediating role, Mendelian randomization

## Abstract

**Objective:**

Although observational studies have proven a correlation between allergic diseases and short stature (SS), the causal relationship remains unestablished. Furthermore, inflammatory proteins are closely linked with both conditions; their potential mediating role has yet to be elucidated. This study aimed to probe into the causal linkage between allergic diseases and SS and the mediating role of inflammatory proteins.

**Methods:**

A two-sample MR analysis was employed to explore the causal links between five allergic diseases (allergic rhinitis, childhood asthma, allergic conjunctivitis, atopic dermatitis (AD), and urticaria) and SS. A two-step MR approach was further used to investigate the mediating role of inflammatory proteins.

**Results:**

Among the five allergic diseases, MR analysis only identified a suggestive positive correlation between AD and the risk of SS (OR = 1.720, 95%CI: 1.081-2.735, *P* = 0.022). A suggestive negative association was also found between AD and the level of the inflammatory protein AXIN1 (OR = 0.922, 95%CI: 0.855-0.995, *P* = 0.037). Mediation analysis revealed that AXIN1 levels potentially mediated 13.5% of the causal pathway between AD and SS risk.

**Conclusion:**

A potential positive causal link between AD and SS was revealed, highlighting the potential mediating role of the inflammatory protein AXIN1. These findings provide a scientific basis and a novel potential target for interventions aimed at improving height growth outcomes in patients with allergic diseases.

## Introduction

Allergic diseases refer to abnormal reactions of the body’s immune system to external substances, including allergic rhinitis, asthma, and atopic dermatitis (AD) [1]. The global incidence of allergic diseases has continued to rise, particularly among children, posing a significant global health challenge [1]. Data from the GBD study show that in 2021, the number of children affected by asthma and AD worldwide was as high as 95.7 million and 72.4 million cases, respectively, with age-standardized prevalence rates of 4,758 and 3,600 cases per 100,000 people [2]. The prevalence of these diseases also imposes a substantial economic burden. Data from the Health Economics Registry for Allergic Diseases indicates that the average annual total cost per patient with allergic diseases reaches up to €1,329.55, of which indirect costs account for €338.68 [3]. Beyond this, the potential broader health impacts of allergic diseases are increasingly receiving attention from the academic community, particularly regarding their negative effects on children’s height.

Children with allergic diseases, whether bronchial asthma, AD, or allergic rhinitis, have a two-to-five times higher rate of short stature (SS) compared to healthy individuals [4]. Further evidence suggests that asthma, especially severe or uncontrolled cases, can temporarily impair children’s growth, leading to the occurrence of SS [5]. However, most of these studies are observational analyses, and evidence clarifying the causal linkage between allergic diseases and SS is still lacking. Notably, the effects of different allergic diseases on height may be heterogeneous. Asthma may impair growth via chronic airway inflammation and airflow limitation [6], whereas AD may affect growth through pruritus-induced sleep disruption, chronic inflammation, and other pathways [7]. Beyond the pathological effects of the diseases themselves, corticosteroid therapy, widely used in clinical practice, also impacts childhood growth. Oral glucocorticoids markedly suppress linear growth; inhaled formulations exhibit low bioavailability, yet long-term administration at moderate-to-high doses can still slow growth velocity [8]. Collectively, the intertwined growth-inhibiting effects of chronic disease-related inflammation and therapeutic medications render the association between allergic diseases and short stature particularly complex, underscoring an urgent need to clarify their causal relationship.

More importantly, the potential factors underlying the linkage between allergic diseases and SS remain to be systematically elucidated. It is well known that allergic diseases are essentially a group of chronic inflammatory conditions. For instance, the core pathological processes of asthma, rhinitis, and AD are all closely linked with dynamic changes in a series of inflammatory proteins [9]. Interestingly, growth impairment frequently accompanies a state of chronic inflammation, with effects ranging from a slight reduction in height velocity to severe SS [10]. Furthermore, experimental evidence indicates that diagnostic models based on inflammatory proteins have achieved a specificity of 91.2% for diagnosing SS [11]. Considering this, the authors hypothesize that inflammatory proteins may be the critical link connecting allergic diseases and SS.

Therefore, this study employs the Mendelian Randomization (MR) method to investigate the potential causal linkage between allergic diseases and SS and further evaluates the potential mediating role of inflammatory proteins. The discovery provides a scientific basis for clinical interventions to improve the growth outcomes of patients with allergic diseases.

## Methods

### Study Design

The flowchart was displayed (Figure 1): (1) A two-sample MR analysis was undertaken to probe into the causal linkage using five allergic diseases (allergic rhinitis, childhood asthma, allergic conjunctivitis, AD, and urticaria) and inflammatory proteins as the exposure factors, respectively, and SS as the outcome variable. (2) The mediating role of inflammatory proteins in the linkage between allergic diseases and the risk of SS was evaluated. To avoid bias due to heterogeneity, all genome-wide association study (GWAS) data were derived from individuals of European ancestry.

**Figure 1:**
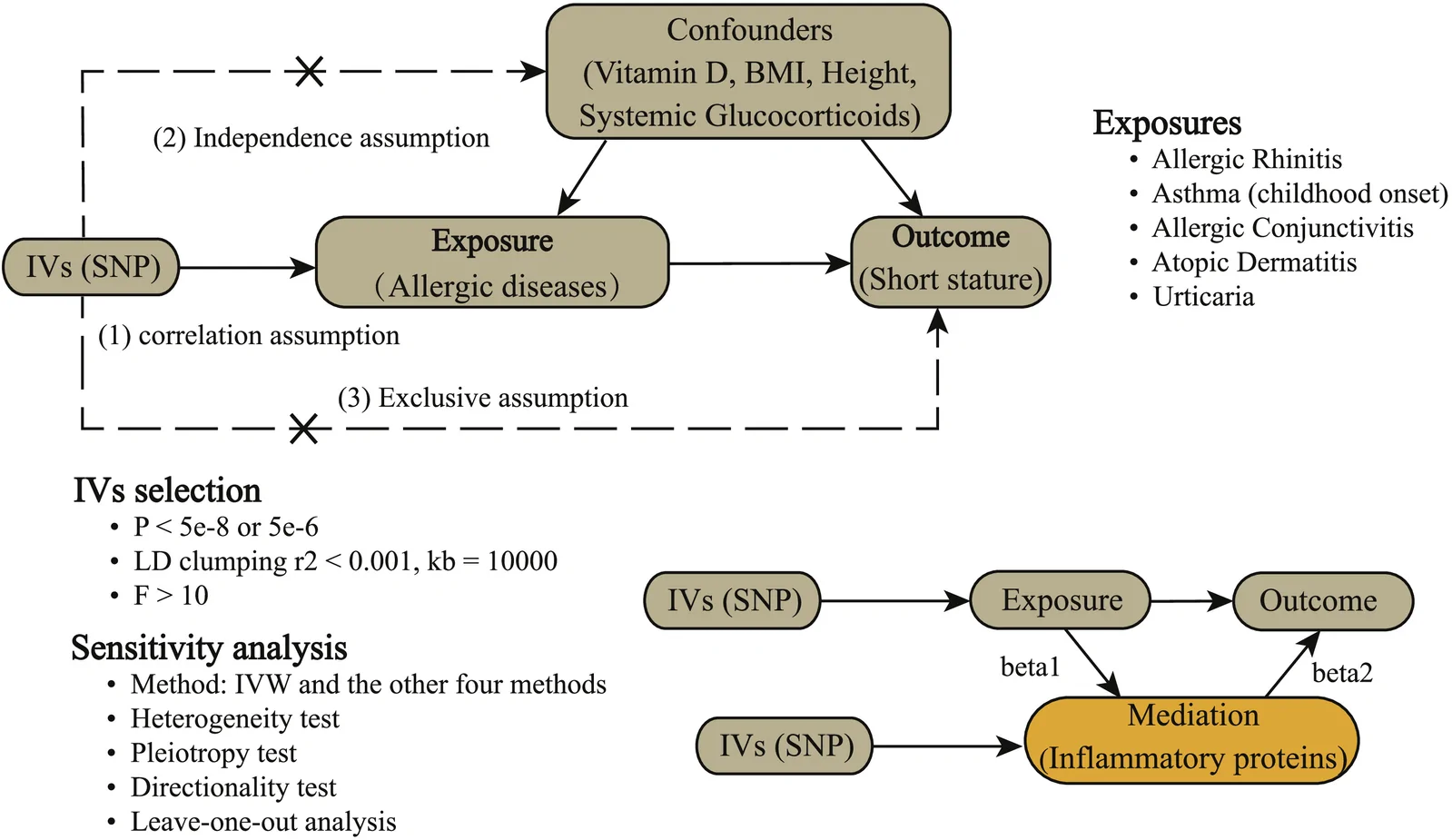
Flow chart of the Mendelian Randomization study design.

### Acquisition of GWAS data

GWAS data for five allergic diseases were included: (1) Participants with allergic rhinitis were from the US Veterans Administration Million Veteran Program, comprising 92,310 cases and 311,377 controls; [12] (2) Childhood asthma data were sourced from the UK Biobank, restricted to individuals with a first disease diagnosis at age ≤ 19 years, totaling 13,962 cases and 300,671 controls; [13] (3) Allergic conjunctivitis data were from the US Veterans Administration Million Veteran Program, involving 438,171 individuals of European ancestry, including 8,602 cases; [12] (4) AD data were obtained from a study by Sliz E et al., [14] including 796,661 European individuals, with 22,474 cases; (5) Urticaria data were from the UK Biobank, comprising 458,440 European individuals, including 10,191 patients [15]. The complete datasets were downloaded from the GWAS Catalog database (https://www.ebi.ac.uk/).

The GWAS data for inflammatory proteins were sourced from 11 cohorts (14,824 European participants). 91 inflammatory proteins were measured with the Olink Target platform inflammation panel (Table S1) [16]. The data are available in the GWAS Catalog (https://www.ebi.ac.uk/gwas/; IDs: GCST90274758 to GCST90274848).

The GWAS data for SS were derived from the Finnish FinnGen database release R7 (https://www.finngen.fi/en) [17], consisting of 298,054 samples, including 464 cases. This outcome was defined as a binary disease phenotype constructed based on diagnostic codes from Finnish national health registries, rather than a quantitative trait derived from continuous height measurements or height Z-scores. In FinnGen, SS was identified using the following International Classification of Diseases, 10th Revision (ICD-10) codes: E34.30, E34.31, E34.35, and E34.39. Accordingly, the present study evaluated associations between genetically predicted susceptibility to allergic diseases and registry-diagnosed SS, instead of continuous height Z-scores. GWAS data are presented in Table 1.

**Table 1:** Detailed information of the GWAS data in the study.

Traits	ID	Population	Sample	Case	Control	PubMed
Allergic Rhinitis	GCST90476021	European	403,687	92,310	311,377	39024449
Asthma (childhood onset)	GCST007800	European	314,633	13,962	300,671	30929738
Allergic Conjunctivitis	GCST90477740	European	438,171	8,602	429,569	39024449
Atopic Dermatitis	GCST90027161	European	796,661	22,474	774,187	34454985
Urticaria	GCST90473943	European	458,440	10,191	448,249	40770095
91 Inflammatory proteins	GCST90274758-GCST90274848	European	14,824	/	/	37563310
Short stature	FinnGen	European	298,054	464	297,590	36653562

### Selection of instrumental variables (IVs)

IVs were selected: (1) Single-nucleotide polymorphisms (SNPs) served as the IVs. For exposures including allergic rhinitis, childhood asthma, and AD (genome-wide significance threshold: *P* < 5 × 10⁻⁸). To ensure an adequate number of SNPs for analysis for each exposure, *P* < 5 × 10⁻⁶ was used for allergic conjunctivitis, urticaria, inflammatory proteins, and SS. Applying the stringent threshold of *P* < 5e-08 would lead to severe loss of statistical power and preclude reliable causal inference. In contrast, the relaxed threshold of *P* < 5e-06 has been widely adopted in existing MR literature, with its validity well recognized. (2) Clumping was performed to exclude SNPs in linkage disequilibrium (r^2^ = 0.001, window size = 10,000 kb). (3) SNPs were matched and screened against known phenotypes (https://www.ebi.ac.uk/gwas/). The authors excluded potentially pleiotropic SNPs correlated with height or other phenotypes that could directly affect SS, including body mass index, standing height, systemic glucocorticoid exposure, and vitamin D status (Table S2). It is worth clarifying that the exclusion of height-related SNPs does not treat height as a conventional confounding factor. Instead, this filtering step aims to minimize direct IV effects on the outcome of SS, thereby preventing violations of the MR exclusion restriction assumption. (4) SNP harmonization was conducted to remove palindromic SNPs with ambiguous strand orientation and incompatible alleles. (5) The F-statistic was calculated to exclude weak IVs; all included SNPs had an F-statistic > 10. (6) MR-PRESSO outlier testing was performed to detect and remove outlier SNPs, thereby correcting for horizontal pleiotropy.

### MR analysis

The random-effects inverse variance weighted (IVW) method served as the primary analytical approach. MR-Egger regression, the weighted median estimator, the weighted mode, and the simple mode were employed to evaluate the robustness of the findings. A *P*-value < 0.05 for the IVW method estimate indicated statistical significance. To examine whether the causal linkage results were influenced by reverse causality, reverse MR analysis was conducted under the same criteria.

The mediation analysis use a two-step MR approach. The causal linkage from allergic diseases to SS constituted the total effect (Total beta). The mediation analysis comprised (1) estimating the causal linkage between the allergic disease and the inflammatory protein (beta1), and (2) estimating the causal effect between the inflammatory protein and SS (beta2). The mediation effect was calculated (beta1 × beta2). The mediation proportion was estimated (mediation effect/total effect). By using the Delta method, the 95%CI for the mediation effect was estimated. In addition, 1,000 parametric bootstrap resampling were performed based on β₁, β₂, and their standard errors to estimate the standard error, 95% confidence intervals, and P values of the indirect effect.

### Sensitivity analysis

Sensitivity analyses were completed with heterogeneity tests, horizontal pleiotropy tests, and “leave-one-out” analysis. Cochran’s Q test was applied to assess heterogeneity among the IVs, with a *P*-value < 0.05 indicating the presence of heterogeneity. Horizontal pleiotropy was examined with MR-Egger regression and the MR-PRESSO global test, with a *P*-value < 0.05 suggesting significant horizontal pleiotropy. To ensure the robustness of the MR findings, results exhibiting horizontal pleiotropy were excluded. The “leave-one-out” analysis was undertaken to verify that the MR results were not disproportionately driven by any single SNP. The Steiger directionality test was applied to assess the direction of the causal linkage, thereby ensuring that the results were not biased by reverse causality.

### Statistical analysis

Analyses were processed with R software (version 4.4.1). The two-sample MR analyses were completed with the R packages “Two Sample MR” (version 0.6.13) and “MR-PRESSO” (version 1.0). Considering the multiple MR tests performed in this study, the Benjamini–Hochberg procedure was applied for false discovery rate (FDR) correction. FDR correction was conducted separately for three sets of analyses: associations between the five allergic diseases and SS, associations between the 91 inflammatory proteins and SS, and associations between AD and candidate inflammatory proteins. Results with FDR < 0.05 were regarded as statistically significant after multiple testing correction; associations with *P* < 0.05 yet failing to pass FDR correction were defined as suggestive correlations.

## Results

### Genetic linkage between allergic diseases and SS

Following the selection criteria, the IVs included for the allergic diseases are detailed in Table S3. The IVW analysis revealed a suggestive positive association solely between AD and the risk of SS (OR = 1.720, 95% CI: 1.081–2.735, *P* = 0.022) (Figure 2A, Table S4). No statistically significant causal associations were detected between SS and the remaining allergic diseases. Specifically, the IVW estimates for other allergic diseases versus short stature were as follows: childhood asthma (OR = 1.006, 95% CI: 0.985–1.028, *P* = 0.570); urticaria (OR = 0.912, 95% CI: 0.568–1.465, *P* = 0.704); allergic rhinitis (OR = 2.814, 95% CI: 0.769–10.294, *P* = 0.118); and allergic conjunctivitis (OR = 1.140, 95% CI: 0.726–1.791, *P* = 0.569) (Table S5).

**Figure 2:**
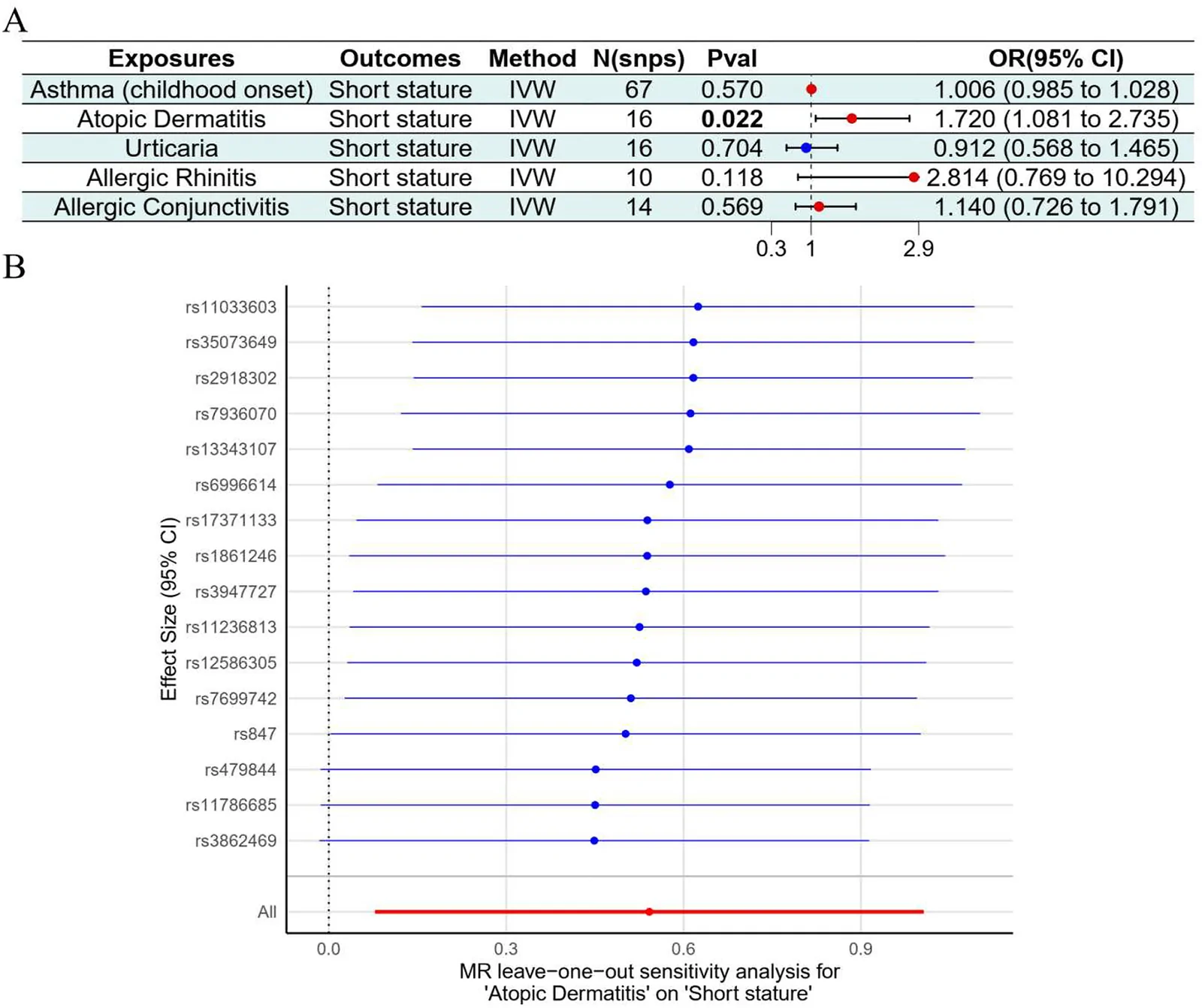
The genetic association results between allergic diseases and short stature. (A). The IVW results between allergic diseases and short stature. (B). Leave-one-out sensitivity analysis of the association between allergic diseases and short stature.

Sensitivity analyses indicated no significant heterogeneity or horizontal pleiotropy in the MR results, and the directionality of the causal relationship was confirmed (Table 2). The leave-one-out analysis demonstrated that no single IV unduly influenced the identified causal association (Figure 2B). To further assess whether the limited number of SS cases might compromise the robustness of the present findings, the authors performed supplementary analyses using a GWAS dataset for continuous height (GCST90475368) as an alternative outcome, which included 283,251 participants [12]. Genetically predicted AD was found to be associated with reduced continuous height (Table S6). This effect direction was consistent with the primary analysis showing that AD increased the risk of SS, implying that genetic susceptibility to AD may correlate with height-related phenotypes. Furthermore, reverse MR analysis, treating SS as the exposure and AD as the outcome, did not reveal evidence of reverse causality (OR = 1.004, 95%CI: 0.983-1.026, *P* = 0.686) (Figure S1). Therefore, the positive linkage between AD and the risk of SS is robust and reliable.

**Table 2:** The sensitivity analysis results of the relationship between allergic diseases and short stature.

Exposures	Outcome	Heterogeneity test				Pleiotropy test				Direction test	
		IVW		MR Egger		MR Egger		MR-PRESSO		Causal	Steiger_pval
		Q	Q_pval	Q	Q_pval	Intercept	Pval	RSSobs	Global pval		
Asthma (childhood onset)	Short stature	83.099	0.076	80.792	0.089	-0.129	0.178	85.660	0.098	TRUE	0
Atopic Dermatitis	Short stature	15.810	0.395	13.073	0.521	0.157	0.120	17.889	0.409	TRUE	1.32E-27
Urticaria	Short stature	13.385	0.573	12.882	0.536	0.043	0.489	15.998	0.517	TRUE	2.14E-22
Allergic Rhinitis	Short stature	8.809	0.455	6.939	0.543	0.124	0.209	11.743	0.408	TRUE	7.53E-24
Allergic Conjunctivitis	Short stature	12.645	0.476	12.401	0.414	-0.033	0.635	14.514	0.509	TRUE	9.90E-19

To evaluate the potential shared genetic architecture across distinct allergic diseases, the authors identified two overlapping SNPs (rs2918302 and rs479844) between AD and childhood asthma, whereas no overlapping IVs were detected between AD and allergic rhinitis, urticaria, or allergic conjunctivitis (Table S7). The authors subsequently re-conducted the MR analysis of AD and SS after excluding rs2918302 and rs479844. The effect direction remained consistent with the primary analysis. The IVW analysis still revealed a suggestive positive association between genetically predicted AD and elevated SS risk (OR = 1.689, 95% CI: 1.042–2.736, *P* = 0.033) (Table S8). Collectively, the association between AD and SS is not driven by SNPs shared between AD and asthma.

### Genetic linkage between inflammatory proteins and SS

After selection, the IVs related to inflammatory proteins are presented in Table S9. The IVW results showed that ARTN (OR = 0.513, 95%CI: 0.276–0.952, *P* = 0.034), AXIN1 (OR = 0.406, 95%CI: 0.184–0.894, *P* = 0.025), CXCL1 (OR = 0.511, 95%CI: 0.298–0.877, *P* = 0.015), and IL-2 (OR = 0.470, 95% CI: 0.270–0.821, *P* = 0.008) were suggestively negatively linked with SS; whereas IL-7 (OR = 2.912, 95%CI: 1.273–6.660, *P* = 0.011) and TNFSF14 (OR = 1.600, 95%CI: 1.095–2.338, *P* = 0.015) were suggestively positively linked with SS (Figure 3A, Table S5 and S10).

**Figure 3:**
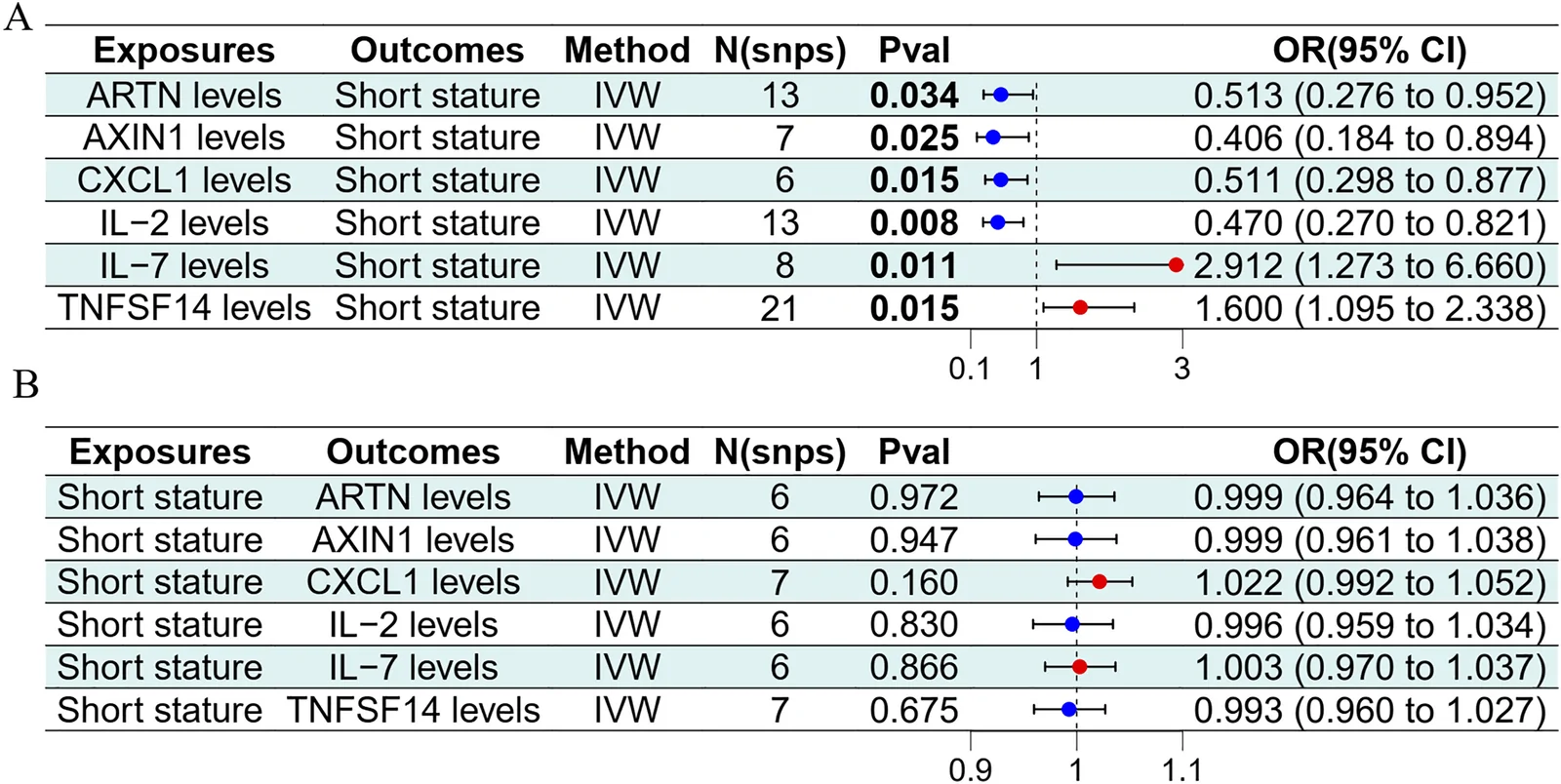
The genetic association results between inflammatory proteins and short stature. (A). IVW Results between inflammatory proteins and short stature. (B). Inverse MR Results between inflammatory proteins and short stature.

Reverse MR analysis indicated no reverse causality in the aforementioned associations (Figure 3B). Sensitivity analyses demonstrated the robustness of the MR results (Table S11, Figure S2). Based on these findings, the authors selected these six inflammatory proteins as potential mediators for further analysis in the relationship between AD and the risk of SS.

### Mediation analysis

The authors further assessed the causal linkage between AD and the six selected inflammatory proteins (Figure S3, Table S5). The results indicated that among the six candidate inflammatory proteins, only AXIN1 exhibited a suggestive genetic association with AD; genetically predicted AD was correlated with reduced AXIN1 levels (OR = 0.922, 95%CI: 0.855–0.995, *P* = 0.037) (Figure 4), a direction consistently supported by other MR methods (Table S12). In sensitivity analyses, the reliability and robustness of these MR results (Table S13, Figure S1) were verified.

**Figure 4:**
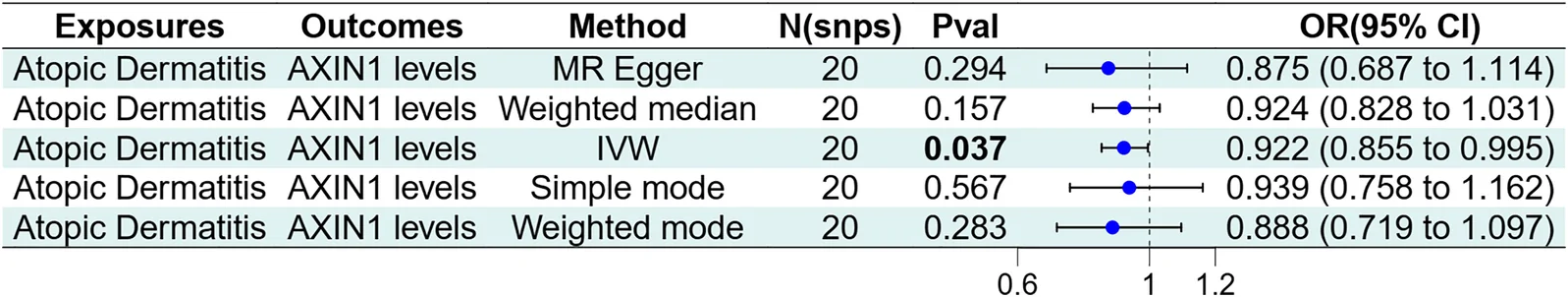
The MR results between atopic dermatitis and inflammatory proteins.

Two-step MR mediation analysis revealed that under the Delta method, the estimated indirect effect of AXIN1 was 0.073, with a corresponding mediation proportion of 13.5% (−3.84%, 30.8%). Further estimation via Bootstrap resampling yielded a mediation proportion of 12.2% (0.3%, 36.5%) for AXIN1, which reached statistical significance (*P* = 0.038). Therefore, AXIN1 may serve as a potential mediator protein underlying the association between AD and the risk of SS (Table 3).

**Table 3:** Mediation analysis result.

Exposure	Mediator	Outcome	Total Beta	Beta1	Beta2	Delta method			Bootstrap method		
						Mediated effect(95%CI)	Mediated proportion(95%CI)	Pval	Mediated effect(95%CI)	Mediated proportion(95%CI)	Pval
Atopic Dermatitis	AXIN1 levels	Short stature	0.542	-0.081	-0.902	0.073 (-0.021, 0.167)	13.5%(-3.84%, 30.8%)	0.127	0.066 (0.002, 0.198)	12.2%(0.3%, 36.5%)	0.038

## Discussion

The present work identified a suggestive positive linkage between AD and the risk of SS. Furthermore, circulating levels of the inflammatory protein AXIN1 were found to potentially mediate this causal relationship.

The observed suggestive positive link between AD and the risk of SS aligns with prior research. For instance, a prospective longitudinal cohort study of 10,611 participants demonstrated that AD was linked with SS in early childhood. Specifically, children with AD were, on average, 0.5 cm shorter at age 2 and 0.6 cm shorter at age 5 compared to their non-affected counterparts [18]. Similarly, another prospective birth cohort study found that persistent eczema (a form of AD) was negatively linked with height at ages 2 and 3 years [19]. These findings collectively underscore that AD, particularly when onset occurs in early childhood, represents a significant risk factor for impaired growth.

Notably, no robust genetic causal evidence was identified for associations between SS and allergic diseases other than AD, which does not rule out the existence of underlying biological links. A more precise interpretation is that, given the currently available GWAS datasets, IV strength and statistical power of the outcome, the authors failed to obtain conclusive genetic evidence supporting the causal effects of these allergic diseases on SS risk. Future studies with larger-scale GWAS data featuring more precisely defined phenotypes, as well as stratification by disease severity, age of onset and treatment status, are required to further validate these null findings.

The exact mechanisms of the suggestive positive link between AD and SS have not been fully elucidated. However, based on existing research, it is likely the result of synergistic effects from multiple factors, including chronic inflammation, sleep disturbances, and therapeutic medications. Chronic inflammation is a central component. Patients with AD exhibit persistent systemic low-grade inflammation, characterized by the release of a large number of pro-inflammatory cytokines (e.g., TNF-α, IL-6) [20]. These cytokines may disrupt the function of the hypothalamic-pituitary growth hormone axis and directly repress the proliferation and differentiation of growth plate chondrocytes, thereby impeding longitudinal bone growth [21]. Furthermore, intense pruritus, a hallmark symptom of AD, often leads to difficulty falling asleep and frequent nocturnal awakenings, causing significant sleep fragmentation [22]. Since growth hormone is primarily secreted in a pulsatile manner during deep sleep stages, reduced sleep quality directly leads to decreased secretion, which can exert a substantial negative impact on height growth over the long term [23]. The influence of therapeutic agents is also a significant consideration. To control the condition, patients with AD often require treatment with topical or systemic glucocorticoids. However, side effects of glucocorticoids can interfere with bone cell metabolism and antagonize growth hormone signaling pathways [24], all of which may increase the risk of SS.

The present study further reveals that circulating levels of the inflammatory protein AXIN1 potentially mediate the link between AD and SS. AXIN1 (also known as Axin) is a highly conserved, multifunctional scaffolding protein in multicellular organisms, most classically recognized as a core negative regulator of the Wnt/β-catenin signaling pathway [25]. Although no studies have directly demonstrated a link between AD and AXIN1, existing evidence indicates that a state of chronic inflammation in the host can influence the expression and function of AXIN1 [26], thereby interfering with the normal transduction of the Wnt/β-catenin axis. It is particularly important to note that the Wnt/β-catenin axis holds an instrumental role in the proliferation, differentiation, and apoptosis of bone cells [27]. Deficiencies in AXIN1-mediated signaling, leading to abnormal activation of β-catenin, have been shown to cause skeletal abnormalities in mice [28]. Furthermore, evidence suggests that dysregulation of the Wnt/β-catenin axis can directly lead to skeletal dysplasia in early human life [29]. Given that impaired skeletal development is closely linked with the risk of SS [30], the authors hypothesize that the dysfunction of the AXIN1-Wnt/β-catenin axis, driven by the chronic inflammation characteristic of AD, may serve as a significant molecular pathway linking AD to SS by affecting bone development. However, this speculation still lacks direct experimental evidence to date. More in-depth and systematic experimental studies are required to confirm the specific role of this molecular pathway.

Therefore, for patients with AD presenting abnormal AXIN1 signaling, the authors propose that the primary and core management strategy is to actively and effectively control disease-related chronic inflammation, so as to alleviate potential disruption of the AXIN1-Wnt/β-catenin axis induced by inflammation. Meanwhile, emphasis should be placed on improving sleep quality and ensuring adequate nutrition to underpin the growth hormone axis and skeletal development. Routine monitoring of serum 25-hydroxyvitamin D concentrations with individualized supplementation is recommended, alongside sufficient calcium intake to maintain a metabolic milieu conducive to bone mineralization.

Certain limitations should be noted. First, the genetic samples were mainly derived from participants of European ancestry. While this selection helps control for population stratification bias, it limits the generalizability of the findings. Due to systematic differences in environmental exposures, genetic architecture, and lifestyle factors among different ethnicities and regions, the genetic link identified here should be extrapolated to African, Asian, or other populations with great caution. Independent replications in non-European ancestry populations are warranted in future studies. Second, although a causal link between AD and SS was revealed by the MR approach, the phenotypic definitions for AD in the current GWAS databases are relatively broad and do not distinguish between disease subtypes (e.g., early-onset vs. late-onset, intrinsic vs. extrinsic). This phenotypic heterogeneity may obscure stronger causal signals within specific subgroups, thereby affecting the identification of heterogeneous mechanistic pathways. Third, the relatively lenient threshold applied for IV selection of certain exposures may introduce bias stemming from weak IVs. The authors performed F-statistic tests and multiple sensitivity analyses throughout the analyses to guarantee the reliability of these results. Fourth, although the number of directly overlapping IVs was limited in this study and the primary findings remained consistent in effect direction after removing shared SNPs, allergic diseases may still share a broader immune genetic background. Accordingly, comorbidity of allergic diseases, disease severity, disease control status, and glucocorticoid therapy, particularly severe or uncontrolled asthma, as well as long-term exposure to inhaled or systemic glucocorticoids, may all affect linear growth. However, this study relied on publicly available GWAS summary statistics without access to individual-level exposure data. Therefore, the authors could not further evaluate the proportion of AD patients complicated by asthma or those receiving steroid treatment, nor conduct stratified MR analyses by mild, moderate, and severe disease. Future studies with individual-level genotypic and clinical phenotypic data can further verify whether the association between AD and SS is independent of comorbid allergic conditions, disease severity, and glucocorticoid treatment. Fifth, the SS outcome adopted in this study was a binary registry-diagnosed phenotype from FinnGen rather than continuous height Z-scores, which led to limited statistical power. Access to individual-level datasets containing continuous height Z-scores, disease severity, and treatment exposure information in future research will facilitate further validation and refinement of the present findings. Finally, despite the statistically significant mediating effect of AXIN1 indicated by Bootstrap analysis, the confidence interval derived from the Delta method crossed zero. Moreover, this mediation analysis was based on two-step MR estimation, which may suffer from insufficient statistical power attributed to weak IV strength, limited sample size, potential sample overlap, multiple testing, and other confounding factors. As such, these results should be interpreted as exploratory and hypothesis-generating findings, requiring further validation using larger, independent proteome-wide GWAS and GWAS datasets related to short stature or height. Additionally, the specific biological mechanisms underlying this mediating effect still require functional validation via experimental studies, such as in cellular or animal models.

## Conclusion

AD is suggestively positively linked with the risk of SS, with circulating AXIN1 potentially mediating the linkage. For patients with AD, in addition to management of skin lesions and pruritus, close attention should also be paid to their height trajectory.

## Funding

No Funding.

## Authors’ contributions

Wei Yin and Liping Wang contributed to data analysis, drafting and revising the article, gave final approval of the version to be published, and agreed to be accountable for all aspects of the work.

## Ethics approval

This study is based on public databases and does not require ethical approval.

## Conflicts of interest

The authors declare no conflicts of interest.

## Data Availability

The data that support the findings of this study are available from the corresponding author upon reasonable request.

## Appendix Supplementary materials

Image, application 1

Image, application 2

Image, application 3

Image, application 4

Image, application 5

Image, application 6

Image, application 7

Image, application 8

Image, application 9

Image, application 10

Image, application 11

Image, application 12

Image, application 13

Image, application 14

## Footnotes

Supplementary material associated with this article can be found in the online version at doi:10.1016/j.jped.2026.101612.
