# Supplementary material for: Causal association between allergic diseases and short stature: a mendelian randomization study revealing the potential mediating role of inflammatory proteins
Source: J Pediatr (Rio J). 2026 Sep 18;103(1):101612. doi: 10.1016/j.jped.2026.101612 (PMC13627808; doi:10.1016/j.jped.2026.101612)
Supplement: Supplementary file 1 [file mmc1.docx]

**JPED-D-26-00118**

**Supplementary Material**

**Figure S1** The MR results between atopic dermatitis and inflammatory proteins, as well as short stature.

**Figure S2** Results of leave-one-out sensitivity analysis between inflammatory proteins and short stature.

**Figure S3** The MR results between atopic dermatitis and six inflammatory proteins.

**Table S1.** Detailed characteristics of 91 inflammation-related proteins.

**Table S2.** Exclude SNPs that are related to height or other phenotypes that may directly affect short stature.

**Table S3.** The instrumental variables included in the phenotypes related to allergic diseases.

**Table S4.** The MR results between allergic diseases and short stature.

**Table S5.** Corrected MR Results.

**Table S6.** The MR results of allergic diseases and inflammatory proteins and height.

**Table S7.** Statistics of overlapping SNPs between allergic diseases.

**Table S8.** The MR results between atopic dermatitis and short stature (excluding SNPs that overlap with allergic diseases).

**Table S9.** The instrumental variables incorporated in the inflammatory protein.

**Table S10.** The MR results between inflammatory proteins and short stature.

**Table S11.** The sensitivity analysis results of the relationship between inflammatory proteins and short stature.

**Table S12.** The MR results between atopic dermatitis and inflammatory proteins.

**Table S13.** The sensitivity analysis results between atopic dermatitis and inflammatory proteins.
